# G protein βγ regulation of KCNQ-encoded voltage-dependent K channels

**DOI:** 10.3389/fphys.2024.1382904

**Published:** 2024-04-09

**Authors:** Jennifer B. Stott, Iain A. Greenwood

**Affiliations:** Vascular Biology Research Group, Institute of Molecular and Clinical Sciences, St George’s University of London, London, United Kingdom

**Keywords:** Kv7, KCNQ, Gβγ, M channel, vasorelaxation

## Abstract

The KCNQ family is comprised of five genes and the expression products form voltage-gated potassium channels (Kv7.1–7.5) that have a major impact upon cellular physiology in many cell types. Each functional Kv7 channel forms as a tetramer that often associates with proteins encoded by the KCNE gene family (KCNE1-5) and is critically reliant upon binding of phosphatidylinositol bisphosphate (PIP_2_) and calmodulin. Other modulators like A-kinase anchoring proteins, ubiquitin ligases and Ca-calmodulin kinase II alter Kv7 channel function and trafficking in an isoform specific manner. It has now been identified that for Kv7.4, G protein βγ subunits (Gβγ) can be added to the list of key regulators and is paramount for channel activity. This article provides an overview of this nascent field of research, highlighting themes and directions for future study.

## Introduction

There are 5 Gβ and 11 Gγ proteins that associate to form a tightly bound dimer, which function as a single entity (Schmidt et al., 1992; Khan et al., 2016). Gβγ subunits associate with Gα subunits (Gα_s_, Gα_i/o_, Gα_q/11_) to form the heterotrimeric G proteins, crucial intermediates for a myriad of cell surface G-protein coupled receptors (GPCRs). However, the 7β propeller structure of Gβ proteins enables interaction with multiple effectors including adenylate cyclases, tyrosine kinases, phospholipases, G-Receptor kinases and MAP kinases (Lin and Smrcka, 2011). In cardiac cells and neurones stimulation of GPCRs coupled to Gα_i/o_ evokes a K^+^ conductance due to the translocation and binding of Gβγ to Kir3.1/3.4 potassium channels, subsequently termed GIRKs (G protein activated Inwardly Rectifying K^+^ channels) (Logothetis et al., 1987; Dascal and Kahanovitch, 2015). Protein biochemistry studies revealed that Gβγ subunits modulate Kir3.1 and Kir3.4 (GIRK1 and GIRK4, respectively) through an interaction with residues 253–348 in the C-terminal as well as residues 41–92 in the N terminus (Huang et al., 1995; He et al., 1999; 2002; Ivanina et al., 2003; Kahanovtch et al., 2014; Touhara and MacKinnon, 2018; Tabak et al., 2019). The molecular determinants of Gβγ binding to GIRKs has been identified by X-ray crystallography (Whorton and MacKinnon, 2013) and is affected by interaction with phosphatidyl inositol bisphosphate (PIP_2_), phosphorylation, scaffold proteins and even Gα_i/o_ proteins (Dascal and Kahanovitch, 2015). Gβγ-dependent activation of GIRKs is a powerful physiological mechanism yet, except for inhibition of Ca_V_2 channels (Herlitze et al., 1996; Ikeda, 1996; Proft and Weiss, 2015) and the recently discovered modulation of TRPM3 channels, examples of Gβγ modulating other ion channels are rare. This article highlights a nascent research field about Gβγ regulation of Kv7 channel voltage-gated potassium channels.

### Kv7 channels

The Kv7 channel subfamily is comprised of five members, Kv7.1–Kv7.5, encoded by the genes KCNQ1 to KCNQ5, which have been identified as key players in controlling excitability and physiological function in many cell types (Barrese et al., 2018). Kv7 proteins have the standard protein topography consistent within the Kv channel family with 6 main transmembrane domains, a pore and selectivity filter created by amino acids between the 5th and 6th domains and a voltage-sensing unit comprised of transmembrane domains one to four with a positively charged 4th domain providing voltage sensing (Coetzee et al., 1999; Ranjan et al., 2019). All Kv7 channels are tetramers with Kv7.1 conventionally forming homotetramers whilst the other Kv7 proteins are capable of heterotetramerisation determined by coiled-coil motifs in the distal C-terminus (Schwake et al., 2003; 2006).

In terms of expression, in the human body Kv7.1 is found in the cochlea and epithelia as well as cardiac myocytes, where it mediates late repolarisation of the action potential. Kv7.2/7.3 channels are robustly expressed in central, peripheral, and sensory neurons, where they underlie a K^+^ conductance known as the M-current crucial for limiting neuronal excitability (Jentsch, 2000; Soldovieri et al., 2011). Kv7.4 is found in the cochlea as well as smooth muscle where it opposes muscle contraction (Greenwood and Ohya, 2009; Haick and Byron, 2016). Kv7.5 is also located in smooth muscle usually in association with Kv7.4 (Brueggemann et al., 2014; Chadha et al., 2014), as well as neurones and skeletal muscle (Barrese et al., 2018). Effective functioning of Kv7 channels is essential for homeostatic processes and when Kv7.1-7.5 channels do not work the consequences can be disastrous. Relatively rare inherited disorders like Long QT syndrome-associated arrhythmia or epileptic encephalopathy as well as more common congenital diseases like atrial fibrillation and non-syndromic deafness are associated with mutations to KCNQ genes (for more details see Barrese et al., 2018; Nappi et al., 2020; Vigil et al., 2020; Huang et al., 2023). Moreover, corruption of Kv7 function has been linked to development of multiple disorders which pose a significant health burden. This includes hypertension, neuropathic pain, urinary incontinence and pre-term labour (see Jepps et al., 2011; 2016; McCallum et al., 2011; Svalø et al., 2015; Carr et al., 2016).

### Kv7 regulation

Kv7 channels are opened by membrane depolarisation due to coupling of the voltage-sensing domain with the pore loop (see Wang et al., 2020 for overview). In addition, Kv7 channel activity is regulated by several modulators (see Haitin and Attali, 2008; Barrese et al., 2018) with PIP_2_ and calmodulin having considerable control over channel activity.

Structure-function studies have identified PIP_2_ and calmodulin binding sites in the distal C-terminus (Haitin and Attali, 2008; Hernandez et al., 2008). Additional PIP_2_ binding sites have been identified at amino acids in S2-S3 and S4-S5 linkers depending upon the Kv7 isoform (Choveau et al., 2018; Brueggemann et al., 2020). The activity of all Kv7 channels is enhanced by PIP_2_ (Li et al., 2005; Hernandez et al., 2008, see Zaydman and Cui, 2014 for a fuller summary), which alters the open probability of Kv7 channels through various mechanisms (see Zaydman and Cui, 2014). Stabilization of the voltage-sensing domain-pore gate coupling is a proposed model for the action of PIP_2_ on Kv7.1 (Sun and MacKinnon, 2020). Calmodulin binds to a site overlapping with the PIP_2_ binding domain on the C-terminus (Haitin and Attali, 2008) and leads to inhibition of Kv7.2/7.3, Kv7.4 and Kv7.5 but stabilises Kv7.1 activity (Gamper et al., 2005; Tobelaim et al., 2017).

The biophysical, pharmacological and trafficking properties of Kv7 channels are also dictated by association with proteins encoded by the KCNE gene family (KCNE1-5, Abbott, 2020; 2022). The best studied of Kv7-KCNE interactions is Kv7.1 and KCNE1, which constitute channel responsible for the slowly activating late component of ventricular and atrial action potential repolarisation (Barhanin et al., 1996). In this case the KCNE1 protein interacts with the voltage-sensor domain and slows channel opening (Nakajo and Kubo, 2007; Abbott, 2022). However, Kv7.1 also associates with KCNE2 and KCNE3 in epithelial cells and here the channel loses time-dependent properties as the voltage-sensor becomes locked by the interleaving of the KCNE proteins (Abbott, 2022). In smooth muscle cells Kv7.4 and Kv7.4/7.5 heteromers associate with KCNE4 (Jepps et al., 2015), which increases membrane abundance and voltage-sensitivity. Different Kv7 channels are also modulated by protein kinase A and protein kinase C that associate with the channel through interactions with A-kinase anchoring proteins (AKAPs) (Haitin and Attali, 2008; Barrese et al., 2018). Consequently, Kv7 channels exist in a multi-protein complex with many interacting modulators.

### Kv7 channels and Gβγ

**FIGURE 1:**
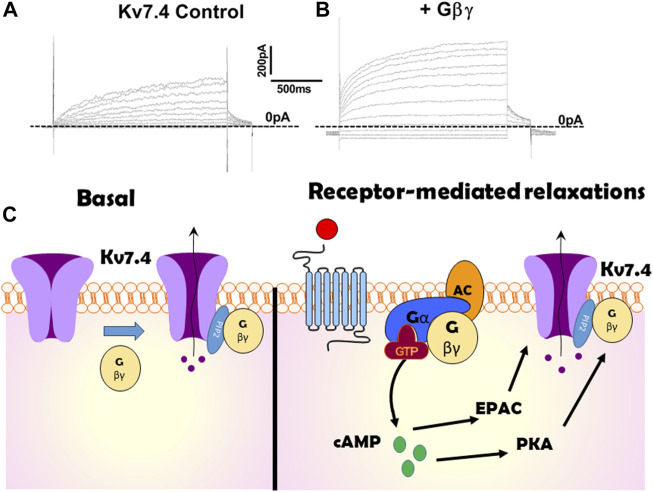
Illustration of the effect of Gβγ on Kv7.4 channels. **(A,B)** show potassium currents generated at different test potentials from −80 mV to + 40 mV in CHO cells expressing Kv7.4 in the absence **(A)** or presence **(B)** of internal perfusion with Gβγ. **(C)** is a cartoon representation of the importance of Gβγ on Kv7.4 channels both at rest (left panel) and in response to receptor-agonists (right). Kv7.4 association with Gβγ is crucial for channel function in many arteries. Gβγ -effector inhibitors suppress heterologously expressed voltage-gated potassium currents in the absence of any receptor stimulation or artificial enrichment. Gβγ association with Kv7.4 is also critical for PKA to enhance channel activity and produce relaxation in some arteries (e.g., renal). However, in other arteries cAMP signalling via EPAC is not reliant upon Gβγ.

In 2015 Stott et al. identified that Gβγ regulates Kv7.4, the Kv7 isoform that is abundant in arterial smooth muscle (Stott et al., 2015b). Intracellular perfusion of active Gβγ isolated from bovine brain augmented the amplitude of potassium currents evoked by membrane depolarization in Human Embryonic Kidney cells (HEKs) constitutively expressing Kv7.4 (see Figure 1 for example). The augmentation took about 5 min to plateau and was associated with an approximate halving of the slow time constant of activation and −5 mV shift in the voltage dependence of activation. Experiments performed with excised patches of cell membrane with the internal surface facing the bathing solution (termed inside out patches) revealed that Gβγ produced a concentration-dependent (0.4–50 ng/mL) increase in open probability with an approximate concentration for half maximal stimulation of 8 ng/mL (Povstyan et al., 2017) without altering the unitary conductance (Stott et al., 2015b; Povstyan et al., 2017), that was identified as 2.3 pS consistent with other studies (Li et al., 2005). Heterologously expressed Kv7.4 channel currents were also augmented with a concomitant reduction in activation kinetics by stimulation of P2Y receptors endogenous to HEK cells with ATP (Stott et al., 2015b).

In a more physiological scenario, native Kv7 currents in freshly dispersed renal artery smooth muscle cells were enhanced by intracellular perfusion with brain derived Gβγ (Stott et al., 2015b). There are five isoforms of Gβ that have differential effects on target proteins (Schmidt et al., 1992; Khan et al., 2016). Co-expression of plasmids containing Gβ1 or Gβ3 in Kv7.4 expressing Chinese Hamster Ovary cells produced an increase in current amplitude, leftward shift in voltage-dependence and reduction in the kinetics of activation that were analogous to the effects of purified brain Gβγ (Greenwood and Stott, 2020). Similar effects were observed with Gβ5, which is structurally dissimilar to the other 4 Gβ isoforms (Khan et al., 2013), but not with overexpressed Gβ2 or Gβ4 (Greenwood and Stott, 2020). Interestingly, molecular knockdown of Gβ3 but not Gβ1 or Gβ5 reduced native Kv7 channel currents in renal artery smooth muscle cells (Greenwood and Stott, 2020). There are 12 genes that encode for Gγ subunits, and the expression products are structurally more diverse than Gβ (27%–76% similarity, Dupré et al., 2009). There is no information about individual Gγ subunits differentially affecting ion channels.

### Constitutive activity-an obligatory role for Gβγ

GIRK channels are modulated by Gβγ subunits at rest (basal activity) and augmented by Gβγ liberated upon activation of receptors coupled to (Kahanovtch et al., 2014). Kv7.4 channels appear to operate in a similar manner but with the basal interaction being more important than with GIRKs. Proximity ligation assays (PLA, Soderberg et al., 2008) with antibodies against Kv7.4 and pan-Gβ revealed considerable association between the two proteins in heterologous expression systems and arterial smooth muscle cells in the absence of receptor stimulation or internal enrichment with Gβγ (Stott et al., 2015b). PLA with antibodies specific for Gβ1 or Gβ3 also exhibited significant basal interaction with Kv7.4 in renal artery myocytes whereas Gβ2 and Gβ4 did not interact. Moreover, structurally different prohibitors of Gβγ-protein interaction not only prevented Kv7.4 current enhancement by Gβγ enrichment but also abrogated Kv7.4 currents. Thus, voltage-dependent K_+_ currents in HEKs constitutively expressing Kv7.4 were reduced to negligible levels after 10min application of the small molecule inhibitors gallein, M199K and M201; a peptide mimetic of the G-protein receptor kinase (Grk2i) and an antibody against Gβ (Stott et al., 2015b; Povstyan et al., 2017). Gallein, M201 and Grk2i also reduced the open probability of Kv7.4 channels recorded in inside-out patches or cell attached recordings to negligible levels (Stott et al., 2015b; Povstyan et al., 2017) and reduced the number of protein-protein interactions derived by proximity ligation assay. These data revealed that the association of Gβγ with the Kv7.4 channel was obligatory for the channel to respond to membrane depolarisation.

### Kv7.4-Gβγ relationship in receptor mediated vasorelaxations

The powerful regulation of Kv7.4 by Gβγ has considerable physiological relevance in arterial smooth muscle both at rest and in the vascular response to receptor-linked vasodilators. In arterial smooth muscle cells Kv7.4 exists predominantly as a heteromer with Kv7.5 (Chadha et al., 2014; Brueggemann et al., 2014). Application of pan-Kv7 blockers like linopirdine or XE991 either produce a contraction, which is sensitive to calcium channel blockers, or sensitizes vasoconstrictor responses. Agents that activate Kv7 channels like ML213, retigabine, maxipost are effective relaxants of precontracted arteries (e.g., Jepps et al., 2015). PLA has revealed a high level of association between Kv7.4 and Gβγ in rat renal arterial smooth muscle cells (Stott et al., 2015b) in the absence of any stimulant. Application of gallein or M199K reduced the number of PLA punctae considerably and caused a marked contraction of the renal artery (Stott et al., 2018), which was equivalent to the effect of a direct Kv7 channel blocker such as linopirdine (Stott et al., 2018). These data suggest that in renal arteries Kv7 channels are important determinants of resting arterial tone that is reliant upon an interaction with Gβγ. In mesenteric arteries there are fewer interactions of Kv7.4 with Gβγ and neither gallein nor Kv7 channel blockers contract the artery. These observations provide credence that Kv7.4-containing channels regulate resting arterial smooth muscle contraction that is reliant upon association with Gβγ but highlight the underlying interaction is complex (Figure 1).

The Kv7.4-Gβγ relationship is also important in receptor-mediated vasorelaxations. In arterial smooth muscle various agonists of Gs-coupled receptors like isoprenaline (mixed β-adrenoceptor) adenosine and calcitonin-gene related peptide, and cGMP stimulants such as atrial natriuretic peptide produce vasodilatation that is impaired if Kv7 channel blockers are present (Chadha et al., 2012; Khanamiri et al., 2013; Stott et al., 2015a; Morales-Cano et al., 2015; Stott et al., 2016; Stott et al., 2018; Mondéjar-Parreño et al., 2019; Baldwin et al., 2022). Similarly, impairment was observed if Kv7.4 or Kv7.5 was reduced by morpholino or siRNA-mediated molecular interference (Chadha et al., 2014; Stott et al., 2018). Interestingly, the nature of the coupling between Gs-linked receptor and Kv7 channels is artery specific. In renal arteries, the β-adrenoceptor-mediated responses are driven via protein kinases A and an associated A-kinase anchoring protein whereas in mesenteric artery isoprenaline-derived relaxations are mediated by EPAC (Exchange Protein Activated by Cyclic AMP) signalling via the downstream mediators Rap1A and Rap2 with Kv7.4 in these vessels. Thus, PKA or AKAP inhibitors attenuated linopirdine-sensitive isoprenaline relaxations of renal artery, but EPAC inhibitors reduced isoprenaline-mediated relaxations of mesenteric artery (Stott et al., 2016).

With respect to Gβγ role in Kv7.4 activity, cell attached recordings from renal artery smooth muscle cells showed that the activity of linopirdine-sensitive K channels was enhanced by isoprenaline in a gallein-sensitive manner (Stott et al., 2015b). Moreover, isoprenaline increased the number of PLA punctae derived from Kv7.4-Gβγ antibodies (Stott et al., 2018). In addition, gallein impaired the isoprenaline-mediated relaxations in renal artery (Stott et al., 2018). Gallein and M199K also prevented calcitonin-gene related peptide-induced relaxations in mesenteric and cerebral arteries (Meens et al., 2012; Stott et al., 2018). Interestingly, whilst isoprenaline-mediated relaxations of mesenteric artery are sensitive to Kv7 blockade they are not sensitive to Gβγ blockers. Thus, while PKA dependent relaxation of renal arteries was sensitive to Gβγ blockade the EPAC-dependent relaxations were not. Finally, myristolated-SRKALNILGYPDYD, which liberates Gβγ without GTP exchange on the Gα (Goubaeva et al., 2003), relaxed precontracted renal arteries in a linopirdine-sensitive manner. Overall, there is considerable evidence that Gβγ association with Kv7.4 is essential for the channel to respond to membrane voltage changes and is a necessary requirement for the channel to respond to receptor-mediated signals. Disabling Kv7.4-Gβγ interactions reduces channel currents and in arteries leads to marked vasospasm and poor response to many receptor-mediated vasodilators. These data presented a new paradigm to regulate arterial relaxation.

### Relationship with PIP_2_

The activity of Kir3.1/3.4 proteins that comprise GIRK channels are regulated by phosphatidyl inositol bisphosphate (PIP_2_) and intracellular sodium levels as well as Gβγ (Petit-Jacques et al., 1999; Wang et al., 2014; Li et al., 2019). Ultrastructural studies have identified that full activation of Kir3.1/3.4 by Gβγ is contingent upon PIP_2_ stabilising an internal gate distinct from a Gβγ binding site (Whorton and Mackinnon, 2011; 2013). Kv7 channels activity is also reliant upon PIP_2_ interaction (Li et al., 2005; Brown et al., 2007; Hernandez et al., 2008). Kv7.4 has the lowest sensitivity of all Kv7 channels to PIP_2_ with an EC_50_ value in excised patches of about 120 uM (Li et al., 2005; Brown et al., 2007; Hernandez et al., 2008; Povstyan et al., 2017). Povstyan et al. (2017) revealed that the stimulatory effect of PIP_2_ on Kv7.4 in excised patches was prevented by prior application of structurally different Gβγ blockers (gallein, M199K, M201 and Grk2i). Strikingly, the stimulatory effect of Gβγ subunits was abrogated by depletion of PIP_2_ levels through activation of phospholipase C linked receptors in the presence of the P-I-3 kinase inhibitor, wortmannin (Povstyan et al., 2017). Affirmation of a cooperative regulation of Kv7.4 by both signal entities was provided by the observation that a sub-efficacious concentration of Gβγ (1 ng/mL), enhanced the action of low PIP_2_ concentrations to maximal levels (Povstyan et al., 2017). These data suggest that the sensitivity of Kv7.4 to PIP_2_ may be dependent on local Gβγ levels and *vice versa*. Thus, channel regulation is dictated by a synergism of Gβγ and PIP2 like the situation for GIRK channels (Dascal and Kahanovitch, 2015; Li et al., 2019).

### Reflections

The observation that Gβγ stimulated Kv7.4 channels was seminal, and the physiological implications are manifold. However, many questions now exist. Importantly, structural information about the site of Gβγ interaction with Kv7.4 is lacking. Moreover, the molecular mechanisms that link Gβγ with enhanced channel activity and the precise role of Gβγ in the modulation produced by protein kinase A and EPACs remain to be defined. In addition, the role of KCNE subunits in Gβγ-mediated regulation has not been assessed. Future research should address these short falls.

Research into Gβγ regulation of Kv channels is in its infancy and mechanistic insight is scarce. The effect of Gβγ on Kv7.4 channels is extremely powerful and appears to be linked to underlying modulatory processes especially PIP_2_-dependent increases in open probability. Information on the other Kv7 subtypes is lacking. As Kv7.2/7.3 heteromers constitute the M-channel that stabilise neuronal membrane potential Gβγ regulation would have a considerable physiological impact. Interestingly, there are various neurodevelopmental disorders that are linked to mutations in the Gβ genes (GNB1 and 2; e.g., Petrovski et al., 2016). Similarly, Kv7.1 in association has a key role in regulating action potential duration in paced cardiomyocytes and a reliance upon Gβγ association would have much impact on cardiac function. The effect of Gβγ on other Kv channels is very limited with data only on Kv1.1 and auxiliary subunit mediated channel inactivation (see Jing et al., 1999; Michaelevski et al., 2002). In contrast to the Kir3. x family that underlie GIRK channels, the Kv family is considerably larger and more complex in terms of modulation by auxiliary proteins. Defining how Gβγ modulate Kv channel activity will be a busy area for years to come.
